# Biochemical and Genetic Testing of GAA in Over 30.000 Symptomatic Patients Suspected to Be Affected With Pompe Disease

**DOI:** 10.1155/2024/6248437

**Published:** 2024-10-22

**Authors:** Sukirthini Balendran-Braun, Ursula Vinatzer, Sandra Liebmann-Reindl, Manuela Lux, Petra Oliva, Stefaan Sansen, Thomas Mechtler, David C. Kasper, Berthold Streubel

**Affiliations:** ^1^Department of Pathology, Medical University of Vienna, Vienna, Austria; ^2^ARCHIMED Life Science GmbH, Vienna, Austria; ^3^Sanofi Genzyme, Amsterdam, Netherlands

**Keywords:** *α*-glucosidase (GAA) enzyme, dried blood spots (DBSs), *GAA* variants, genotype–phenotype, Pompe disease

## Abstract

Pompe disease (PD) is a rare autosomal recessive lysosomal disorder caused by loss-of-function of the *α*-glucosidase (*GAA*) gene. The deficient GAA enzyme activity may result in potential life-threatening muscle weakness, thus requiring a rapid diagnosis to initiate therapeutic interventions. In this large retrospective study, we analyzed 30.836 PD suspect samples from 57 countries using a two-step approach utilizing dried blood spots (DBSs): biochemical testing of GAA activity followed by complementary genetic sequencing of *GAA* in biochemically conspicuous cases. Of these 30.836 samples, 2% (*n* = 639) were excluded; accordingly, this study consisted of 30.193 cases. Biochemical testing of GAA enzyme activity showed normal values in 28.354 (93.90%) and enzyme activity below the cut-off in 1843 (6.10%) cases. These biochemically suspicious cases were genetically analyzed. We identified 723 Pompe cases with 283 different *GAA* alterations, and 98 variants have been unpublished so far. The most common variant was the splice variant c.-32-13T>G (IVS1). Looking at the IVS1-genotype, the majority was compound heterozygous (*n* = 169) and identified in late-onset cases (*n* = 162). Comparison of early- versus late-onset cases to evaluate whether certain genotypes correlate with the age of onset revealed that homozygosity was predominantly found in infantile (85.65%) and compound heterozygosity in late-onset (76.9%) cases. Analysis of homozygous cases revealed 61% nonsense variants in the early stages and 87% missense variants in the late stages. Mapping of disease-associated (homozygous) missense variants to functional GAA protein domains showed that missense variants were found throughout GAA, but we identified enrichment in the catalytic domain. A strict genotype–phenotype correlation cannot be established; nevertheless, a phenotypic implication of some *GAA* variants could be drawn (e.g., c.896T>C/p.L299P, c.2015G>A/p.R672Q, and c.-32-13T>G). The combined enzyme activity and genetic testing from DBS cards can reliably identify PD and significantly accelerate diagnosis. We identified new genetic variants that contribute to the spectrum of pathogenic variants of the *GAA* gene.

## 1. Introduction

Pompe disease (PD) or Glycogenosis Type II (GSD II; OMIM #232300) is a rare autosomal recessive lysosomal disorder caused by pathogenic variants in *α*-glucosidase (*GAA*). Deficient activity of the GAA enzyme can lead to lysosomal glycogen storage, loss of lysosomal function, and finally cell and tissue damage [1]; the most affected tissues are the heart, skeletal and smooth muscle, plus the nervous system [2, 3]. Based on the age of onset of symptoms, the disease is classified into two subtypes [4]: infantile-onset PD (IOPD), where symptoms arise before 12 months of age with clinically significant cardiomyopathy, and late-onset PD (LOPD), with typical onset after 12 months and characterized by proximal muscle weakness and respiratory insufficiency [5], without significant cardiac involvement. Generally, PD has a reported incidence of 1:40.000 in a Caucasian population [6], where it varies depending on ethnicity and geographic region. For example, in the East Asian population (1:12.125), Ashkenazi Jewish (1:22.851), or African-Americans (1:26.560), a higher incidence is reported [7]. Furthermore, data collected with newborn screening (NBS) programs in different parts of the world (Italy, 1:18.795; Taiwan, 1:18.436; Pennsylvania, 1:16.095; and New York, 1:20.190) show higher incidences than the traditional estimate of about 1:40.000 [8]. Data emerging from these programs is revising our knowledge of the incidence of PD [9]. PD is a challenging disease due to the high variability for the age of onset, affected organ systems, severity of the disorder [4, 10], and overlap of signs and symptoms with other more common neuromuscular disorders. Due to autosomal recessive inheritance, patients with PD have one variant in homozygosis or two different variants in compound heterozygosis [11]. The mutational spectrum of *GAA* is very heterogeneous, and genetic variants are often private, only found in a single-family or in a small population [11–13]. For this reason, until now, more than 600 disease-causing variants have been described in the acid maltase gene (*GAA*) on Chromosome 17.

However, an exception is represented by the c.-32-13T>G splice variant (IVS1) that is very common in patients of Caucasian origin [14]. This leaky splicing variant allows the generation of low levels of normal enzyme [10] and is, in most cases, associated with a second variant in the other *GAA* allele, which is usually more severe [11].

Current standard-of-care treatment options include enzyme replacement therapy (ERT) with recombinant human GAA (rhGAA) [15]. An early diagnosis is of utmost relevance because starting ERT before symptoms lead to optimal outcomes. ERT changes the natural course of the disease in infants, results in much longer survival [10], reduces the need for ventilation, and enhances the patient's quality of life [16].

In this study, we tested 30.836 symptomatic cases using a two-step approach in dried blood spots (DBSs): (I) biochemical testing of GAA activity and (in biochemically conspicuous cases) and (II) complementary genetic sequencing of *GAA*. On the basis of a great number of multiracial samples, we show that the combined testing in our single center is reliable, efficient, and robust. Additionally, we identified new genetic variants that contribute to the spectrum of pathogenic variants of the *GAA* gene. Furthermore, a genotype–phenotype correlation could be drawn for several variants of *GAA* from the data obtained.

## 2. Material and Methods

### 2.1. Study Population

Between May 2013 and February 2021, 30.836 symptomatic cases with informed consent were tested for GAA deficiency by tandem mass spectrometry (MS) using DBSs. After the exclusion of samples due to poor sample quality (e.g., insufficient blood saturation on the DBS card) or ambiguous sequencing results, this retrospective study consisted of 30.193 cases from 57 different countries. IOPD is generally defined as PD that arises before 12 months of age. As clinical data were not comprehensive in all our cases (we do not have information on whether a cardiac evaluation was performed at the time of sampling), we classified into the following two groups: early-onset Pompe cases consisting of infants ≤ 1 year old and late-onset Pompe cases including individuals > 1 year. All cases were biochemically and genetically tested in a single center (ARCHIMEDlife; http://www.archimedlife.com/). Cases from family screening were excluded. Subjects with known *GAA* pseudodeficiency variants were excluded. DBS cards were used in all cases for biochemical and genetic analysis. EDTA blood was requested and genetically analyzed in addition to some sporadic cases. Biochemical testing was regarded as conspicuous if the value was below the cut-off or borderline (see Section 2.2). Genetic analysis was performed in all these biochemically suspicious cases.

### 2.2. Biochemical Testing of GAA Enzyme

The DBS cards were stored at room temperature prior to analysis. Duplicates of 3.2 mm DBS punches were incubated with enzyme-specific buffer solutions containing enzyme-specific substrates and known concentrations of isotopically labeled internal standard analogs of the formed products. The samples were incubated at 37°C for 18–20 h. The reaction was quenched by adding an organic precipitation solution. After centrifugation, the supernatant was analyzed by means of ultra-high performance liquid chromatography tandem MS (UHPLC-MS/MS). The average response ratios between the product signals and the signals of the corresponding internal standard of the duplicates were multiplied with the internal standard concentration in the reaction and standardized to the blood volume (3.2 mm punch equals ~3 *μ*L whole blood) and incubation time, resulting in a turnover measure given as micromoles per liter per hour. A multiplex assay was applied for simultaneous analysis of several lysosomal enzyme activities (ABG-Gaucher disease, ASM-Niemann-Pick A/B disease, GLA-Fabry disease, GAA-PD, and GALC-Krabbe) from DBSs. Biochemical testing of GAA was considered conspicuous if the values were below the established cut-off (3.3 *μ*mol/L/h until June 2019 (1st assay), subsequently 2.0 *μ*mol/L/h (2nd assay) due to a new diagnostic assay) or borderline. The cut-off value was established by a clinical reference value study utilizing > 1000 random samples from different age groups and available samples from genetically confirmed positive samples. The receiver operating characteristic (ROC) approach was used to set cut-off ranges. ROC values were adapted according to the corresponding allowable interrun precision limits.

External quality control (EQC) material provided by the CDC (Centers for Disease Control and Prevention, Atlanta, GA, United States; https://www.cdc.gov/labstandards/nsqap.html) has been used in a continuous EQC scheme to monitor interrun repeatability and interlaboratory comparability throughout the study period.

### 2.3. Genetic Testing

Genomic DNA isolation from DBS cards was performed on the Chemagic 360 Instrument (PerkinElmer) with the isolation kit “Chemagic DNA Blood Spot 3 mm Kit” according to the manufacturer's instructions. Subsequent Sanger sequencing of the entire coding region and flanking intronic regions of *GAA* was performed following standard procedures. As mentioned above, DBS was used in all cases for genetic analysis. In cases with unclear results of Sanger sequencing, a second DBS card was requested along with EDTA blood to perform further extensive genetic workup. The testing of family cases for the confirmation of compound heterozygosity was not included in our study population.

Identified potential pathogenic variants were compared with PubMed (https://pubmed.ncbi.nlm.nih.gov), HGMD [17], ClinVar (https://www.ncbi.nlm.nih.gov/clinvar/), and the PD GAA variant database [18]. Unpublished variants were interpreted according to the ACMG/AMP 2015 guidelines [19].

### 2.4. Statistics and Graphical Tools

The positive predictive value (PPV) was calculated from a 2 × 2 contingency table. Subgroup comparison on means was performed with a one-way ANOVA. The threshold for statistical significance was set at a *p* value of less than 0.05.

The lollipop plots were generated using MutationMapper [20, 21] and modified subsequently. The protein structure of human GAA is adapted from [22, 23].

## 3. Results

### 3.1. Sample Characteristics and General Outcome of Biochemical and Genetic Analyses

In total, 30.836 cases were biochemically analyzed. Six hundred thirty-nine cases (2%) were excluded due to poor sample quality. Consequently, this study comprises 30.197 cases. The median age was 31 years, and the age range was 0–95 years. The total cohort consisted of 13.521 (44.78%) females and 16.676 (55.22%) males.

**Figure 1:**
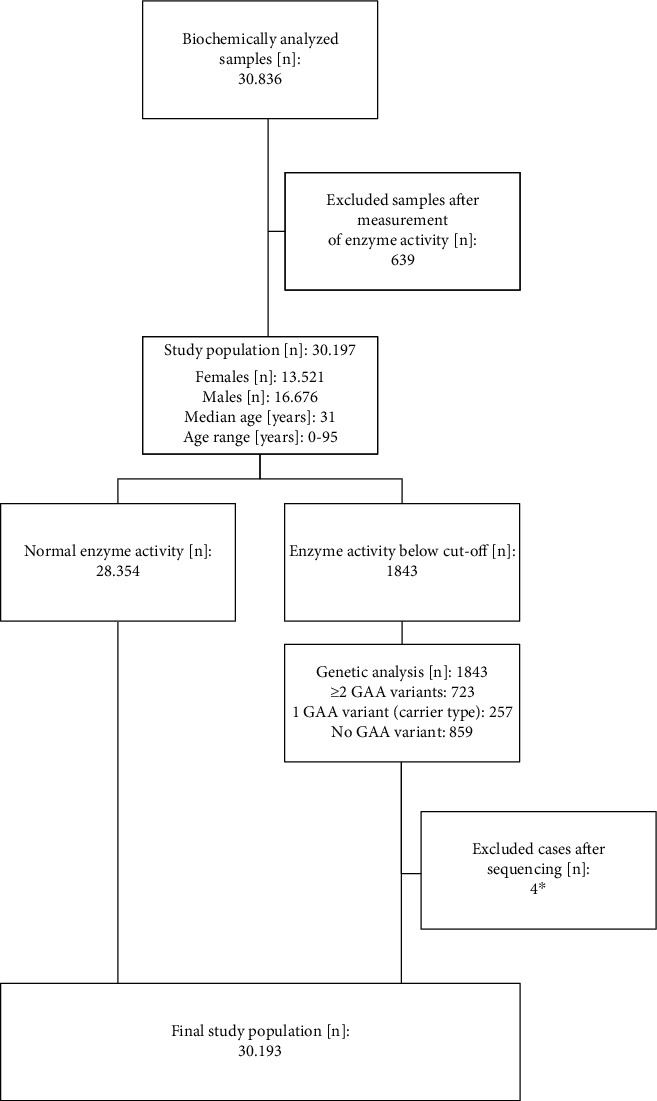
Flowchart of the study population. ⁣^∗^In four cases, the enzyme activity revealed borderline values, and the variants were classified as variants of unknown significance (VUS). Because no further workup was possible, these four cases were excluded.

Biochemical testing of GAA enzyme activity showed normal values in 28.354 (93.90%) cases and enzyme activity below the cut-off in 1843 (6.10%) cases. These biochemically suspicious cases (*n* = 1843) were genetically analyzed. Genetic testing was successful in all samples, with the following results: Among the 1843 cases with conspicuous biochemical results, 723 cases (39.23%) revealed two pathogenic variants. In four cases (0.22%), the enzyme activity revealed borderline values, and variants were classified as variants of unknown significance (VUS). Because no further workup was possible, these four cases were excluded from our cohort analysis for reasons of simplification (Supporting Information 2). In the remaining 1116 cases (60.55%), PD was not confirmed. All results are clearly mapped in Figure 1.

The PPV is defined as the fraction with a positive screening test result that indeed does have the disease. In our total cohort, the PPV of the GAA enzyme activity analysis was 39%. When considering the age of the cases and dividing the cohort into the following two subgroups, a striking difference in the PPV was observed: a PPV of 71% in infant cases and a PPV of 22% in late-onset cases.

### 3.2. Mutational Spectrum of the PD Cohort

**Figure 2:**
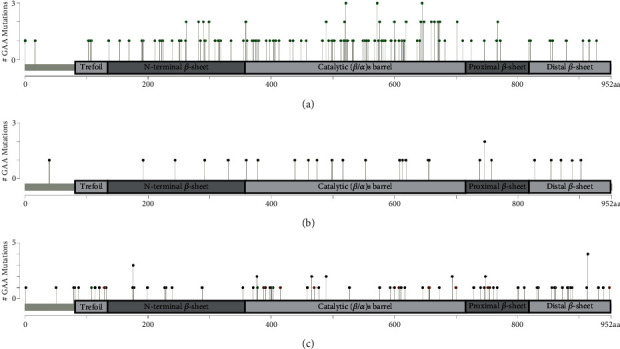
Schematic representation of the found (a) missense variants, (b) stop point variants (nonsense), and (c) frameshift/small in-frame variants along the GAA protein. Any position with a variant obtains a circle. The missense variants are shown in green, truncation variants (stop point, frameshift) in black, and in-frame variants in brown. The thin grey bar represents the entire protein with the different amino acid (aa) positions. The light and dark grey boxes are specific functional domains. The structure of human GAA is adapted from [22, 23]. For reasons of simplification, gross deletions, splice variants, and major in-frame deletions were not shown in this figure.

Next, we analyzed the mutational spectrum of the 723 Pompe cases. A total of 283 different *GAA* alterations were identified and consisted of 138 missense variants, 29 stop point variants, 73 frameshifts (deletions, insertions, and InDels), 29 splicing variants, 10 in-frame deletions, and four gross deletions.  Figure 2 shows a schematic representation of localization of the found missense variants (a), stop point variants (b), and frameshift variants together with small in-frame deletions (c). According to the HGMD database (2023.3), ClinVar, and the PD GAA variant database, 185 of 283 variants were published and 98 variants were unpublished. The unpublished variants were composed of 57 truncation variants/small in-frame variants (Supporting Information 2) and 41 missense variants (Table 1). Twenty-four of the 41 missense variants showed a missense change at an amino acid residue where a different pathogenic missense change has been published before. 65.85% of the 41 unpublished variants were located in the catalytic (*β*/*α*)_8_ barrel domain, a hot spot domain [1]. Pathogenicity of all missense variants is based on the severity criteria provided in the ACMG guidelines for the interpretation of sequence variants [19], taking into account the (I) patient's phenotype, (II) absence in the population, (III) location in a mutational hot spot or well-studied functional domain, and (IV) computational evidence supporting a deleterious effect together with functional information about diminished enzyme activity after repetitive testing.

As aforementioned, 257 cases with one variant (carrier type) were identified in our cohort (Figure 1). In total, 95 different variants were found in these carriers (data not shown). About 45% (43 of 95) of these pathogenic variants were also found in our PD patients. The most frequent variant found in unaffected carriers was the splice variant IVS1 (*n* = 96) with 37%, followed by c.307T>G (*n* = 9), c.2662G>T (*n* = 7), c.1655T>C (*n* = 6), and c.525delT (*n* = 5).

#### 3.2.1. Correlation of Genotypes With Age of Onset in Our PD Cases

**Figure 3:**
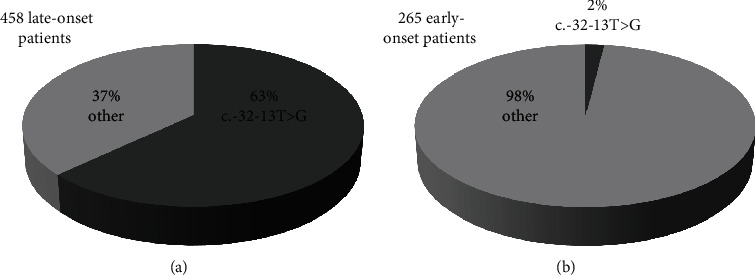
Pie charts showing the percentage of patients with PD ((a) late-onset and (b) early-onset) identified with IVS1 variants. The cases in which we identified IVS1 homozygously or heterozygously were included in this figure.

The most common variant in the entire cohort was the splice variant c.-32-13T>G (IVS1). This alteration was identified in 176 of 723 PD cases. Looking at the IVS1-genotype, the majority were compound heterozygous (*n* = 169), and the remaining seven cases were homozygous. Involving age of onset, IVS1 was identified in 162 compound heterozygous late-onset cases, five homozygous late-onset cases, and some single early-onset cases (*n* = 9) (Figure 3). The c.-32-13T>G variant was found most frequently with c.525delT, c.307T>G, c.2482_2646del (Ex18del), c.784G>A, and c.2662G>T. The mean enzyme activity in these 162 compound heterozygous cases was negligible (0.36 ± 0.33 *μ*mol/L/h). The mean GAA enzyme activity in our total late-onset cases (*n* = 265) was very similar (0.35 ± 0.35 *μ*mol/L/h), and the mean GAA enzyme activity in our infant Pompe cases (*n* = 458) was significantly lower (0.16 ± 0.23 *μ*mol/L/h) (*p* < 0.001; one-way ANOVA).

In the next step, we compared early- versus LOPD cases to evaluate whether certain genotypes correlate with the age of onset. Homozygosity was found predominantly in infantile cases (85.6% homozygous vs. 14.4% compound heterozygous). In contrast, compound heterozygosity is dominant in late-onset cases (76.9% compound heterozygous vs. 24.1% homozygous) (Table 2). A total of 196 different infantile *GAA* alterations and 142 different late-onset *GAA* alterations were found. 58.7% of early-onset alterations presumably result in a premature stop codon, whereas 60.6% of late-onset alterations are missense variants. Several pathogenic *GAA* variants (*n* = 144) were exclusively found in our infantile cases (Supporting Information 2). Likewise, 90 *GAA* variants were found exclusively in late-onset cases (Supporting Information 2). The most frequent alterations among our patients are listed in Table 2.

#### 3.2.2. Homozygous Pompe Cases

**Figure 4:**
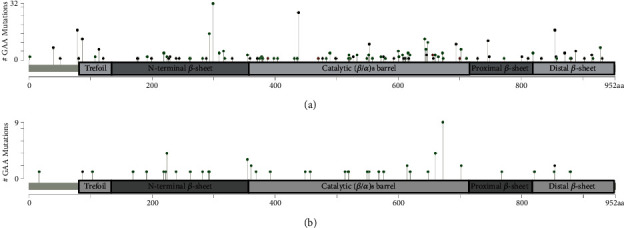
Lollipop plot illustrating all GAA variants in our (a) homozygous early-onset cases and (b) homozygous late-onset cases along the protein structure. Any position with a variant obtains a circle; the length of the line depends on the number of variants detected at that codon. The missense variants are shown in green, truncation variants (stop point and frameshift) in black, and in-frame deletions in brown. The thin grey bar represents the entire protein with the different amino acid (aa) positions. The light and dark grey boxes are specific functional domains. The structure of human GAA is adapted from [22, 23]. For reasons of simplification, gross deletions, splice variants, and in-frame deletions were not shown in this figure.

In autosomal recessive diseases, the pathogenicity assessment of variants is more difficult in compound heterozygous cases than in homozygous cases. Therefore, we focused on cases with homozygous variants. As shown in Table 2, we identified 392 homozygous early-onset and 64 homozygous late-onset cases.  Figure 4 shows the localization of all alterations found in early-onset (a) and late-onset subjects (b). In total, 150 different variants were identified in our homozygous infant cases and consisted of 59 missense variants (39%) and 91 other variants (nonsense variants, splicing variants, deletions, insertions, and InDels). In homozygous late-onset cases, 41 different *GAA* variants were identified, and they were predominantly missense variants (87%). The most frequent alterations were c.896T>C/p.L299P (*n* = 32) in infants and c.2015G>A/p.R672Q (*n* = 9) in late stages, respectively (Table 3).

Next, we compared the localization of early- with late-onset missense variants. Similar frequencies were found for the two predominant domains: catalytic (*β*/*α*)_8_ barrel domain (67% vs. 53%) and N-terminal *β*-sheet domain (21% vs. 30%) (Supporting Information 1). Then, we filtered for shared missense variants in both subgroups and found 16 out of 101 (16.16%) overlapping variants. Furthermore, we performed similar analyses with the identified stop point variants to figure out if some protein domains are more predisposed to alterations than others. Compared to missense variants, stop point variants tend to be more evenly distributed throughout the *GAA* gene (Supporting Information 1).

## 4. Discussion

To our knowledge, this is one of the largest studies with more than 30.000 symptomatic cases suspicious for PD from 57 countries. We performed a two-step approach using DBS cards, which turned out to be fast, reliable, and robust with few dropouts (2%). Biochemical testing of GAA enzyme activity showed reduced activity in 1843 (6.10%) cases and normal values in 28.354 (93.90%) cases. It seems probable that in these cases with normal values, another disorder causes the symptoms. Among the 1843 cases with conspicuous biochemical results, in 1116 cases (60.55%), no PD was confirmed. It is well known that the use of enzyme assays alone can lead to many false-positive cases [9], as implied by the PPV of 39% for the GAA enzyme activity analysis. However, in combination with a second-tier test (here genetic testing), confirmed PD cases with two mutations can be differentiated from carriers with one mutation (*n* = 257) and cases with no *GAA* mutations. It is noteworthy that reduced enzyme activity in carriers is not uncommon [24, 25]. The identification of carriers requires genetic counseling, as there is a much higher risk of having children affected by PD. This was also stated in our reports. However, the results of these family screenings were explicitly excluded from our study of symptomatic cases (see Section 2.1). In total, we identified 723 Pompe cases with 283 different *GAA* alterations. Of these 283 variants, 98 variants have been unpublished so far. Analysis of homozygous cases revealed 87% missense variants in late-onset cases and 61% nonsense variants in early-onset cases.

For several reasons, a fast and reliable diagnosis of PD is crucial. The diagnosis of this life-threatening disease has a major emotional impact on patients and their families [4]. Starting ERT as early as possible and before symptoms have progressed leads to optimal outcomes, and the natural course of the disease in infants results in much longer survival [10], reduces the need for ventilation, and improves the patient's quality of life [16]. Early initiation of ERT is also beneficial for patients with LOPD, particularly those with less advanced disease [26].

Classic IOPD is rapidly progressive, and NBS is an optimal approach for early detection. However, NBS for PD is controversially discussed because the diagnosis of late-onset disease in infancy creates presymptomatic “patients-in-waiting” [27], resulting in anxiety, frustration, and fear of the unknown. In addition, rigorous long-term follow-up is needed to evaluate the best time to start therapy [8]. Furthermore, genetic screening can detect VUS [28]. This may also be problematic in PD because many variants are unique or specific to families [14]. In line with this, 98 new variants were found in our cohort. The classification of these variants was relatively unambiguous according to the ACMG guideline, combining genetic results with zero enzyme activity in repetitive testing in symptomatic cases. The situation is more complex in cases with borderline enzyme activity. The number of such unclear cases (*n* = 4) in our cohort was very low compared to other published studies [29, 30].

The diagnosis of early-symptomatic PD patients is critical due to the high variability in age of onset. A differentiation between both subtypes is not possible on the basis of the enzyme activity level in our cohort. Although the median values of the GAA enzyme level were similarly low for both groups [31, 32], they were statistically different. However, it is not always possible to utilize GAA levels to identify PD status in individuals due to overlapped GAA values [8, 33]. It is well established that the common splice variant c.-32-13T>G (IVS1) is predominantly associated with LOPD [34, 35]. With 63%, IVS1 is by far the most common pathogenic variant in our late-onset subgroup (*n* = 162 compound heterozygous and *n* = 5 homozygous). Although IVS1 is predominantly associated with late-onset cases, a few infant cases with this pathogenic splice variant were identified in our cohort (Table 2 and Table 3). Similar results were published by Rairikar et al. [36]. Bergsma et al. identified the *GAA* c.510C>T variant as a genetic modifier that accelerates symptom onset in compound heterozygous and homozygous IVS patients by lowering enzyme activity [35]. We were unable to confirm this observation because the few infant cases with IVS were all negative for this SNP (data not shown in the Results section). The c.-32-13T>G variant was most frequently found to be associated with c.525delT and c.2482_2646del (Ex18del) on the second allele in our late-onset cases, which is consistent with previous studies [35, 37]. These two variants are quite common in PD [38, 39] and are considered severe [14, 39–41]. Therefore, we conclude that the presence of IVS (milder variant) seems to be sufficient to prevent the occurrence of the severe classic infantile phenotype [14] in the vast majority of cases, irrespective of a second severe variant.

Homozygous patients are readily informative for genotype–phenotype analysis because they carry identical variants on both alleles. Therefore, we focused on our homozygous cases to establish a genotype–phenotype correlation, and the following phenotypic implications of some *GAA* variants could be drawn. The *GAA* variant c.896T>C/p.L299P was identified exclusively in our homozygous early-onset cases, whereas c.2015G>A/p.R672Q was predominantly identified in homozygous late-onset cases (Table 3). The mapping of disease-associated homozygous missense variants to functional GAA protein domains (Figure 4 and Supporting Information 1) showed strong enrichment in the catalytic domain, which is in line with several publications [1, 18, 23, 42, 43]. Based on prediction programs, Thirumal Kumar et al. have shown that deleterious variants in the catalytic domain tend to decrease the tendency to chaperone-binding and/or protein stability [13], which underlines the importance of this domain for GAA enzyme activity. Apart from a genotype–phenotype correlation, the determination of the cross-reactive immunological material (CRIM) status is a relevant parameter for ERT. CRIM-negative IOPD patients typically develop sustained high antibody titers to ERT that render the treatment ineffective [44, 45]. Bali et al. have shown that most CRIM-negative patients were homozygous or compound heterozygous for nonsense variants, frameshift variants, and multiexon deletions [44]. Based on our rapid two-step approach in DBS cards, at least in homozygous infant cases with premature stops or frameshift variants, an immediate prediction of a negative CRIM status may be possible.

Due to the rarity of PD, it is often misdiagnosed for more common disorders with similar symptoms [46]. Some of these disorders are Werdnig–Hoffman disease, Danon disease, facioscapulohumeral dystrophy (FSHD), Duchenne muscular dystrophy, or Becker muscular dystrophy. The awareness of PD should be raised among physicians because PD requires immediate attention to maximize the potential benefit of therapy and prevent irreversible organ damage [4, 46].

One major limitation of our study is the classification of PD patients according to missing clinical data. The request to analyze samples derived from many different physicians from all over the world. Due to partially incomplete request forms, the present study lacks knowledge about the entire clinical details of the analyzed patients, such as cardiac involvement. Given the retrospective nature of our investigations, we were unable to collect the missing data. Therefore, classification of patients in IOPD and LOPD per definition was not possible, so instead, we defined the two subgroups “early-onset PD” and “LOPD.” Although age of onset alone is not an ideal criterion for the classification of PD patients, it does not always delineate subtypes well [4]. Rather, the presence of cardiomyopathy in patients under 1 year of age is the distinguishing feature [30]. Nevertheless, the presented data are useful for the medical community, for example, to assist in diagnostic practice.

## 5. Conclusion

In summary, our two-step approach in DBS cards is recommendable for the identification of PD patients. We were able to identify patients with PD at a very early stage, which can be decisive for ERT because any delay can negatively affect treatment outcomes. Furthermore, the mutational spectrum of our cohort complements the deleterious *GAA* variants responsible for the PD registered so far. As with most genetic diseases, a strict genotype–phenotype correlation cannot be established [1, 14], but a phenotypic implication of some *GAA* variants could be drawn. Furthermore, our cases were multiracial because they were from 57 countries, so they can be considered very representative without ethnical biases.

## Figures and Tables

**Table 1:** Novel identified missense variants.

**No.**	**Variant**	**Amino acid**	**Protein domain**	**N**		
Similar variants were already published					Variant	Published amino acid
1	c.786G>C	p.E262D	NTB	1	c.784G>C/c.784G>A	p.E262Q/p.E262K
2	c.868A>C	p.N290H	NTB	1	c.869A>T	p.N290D
3	c.868A>G	p.N290D	NTB	1	c.869A>T	p.N290D
4	c.874T>C	p.Y292H	NTB	1	c.875A>G	p.Y292C
5	c.953T>G	p.M318R	NTB	1	c.953T>A/c.953T>C	p.M318K/p.M318T
6	c.1493G>C	p.W498S	CCB	1	c.1493G>C	p.W498⁣^∗^
7	c.1559A>T	p.N520I	CCB	3	c.1559A>G	p.N520S
8	c.1562A>G	p.E521G	CCB	1	c.1562A>T	p.E521V
9	c.1646G>T	p.G549V	CCB	2	c.1645G>A/c.1645G>C	p.G549R
10	c.1673G>A	p.C558Y	CCB	1	c.1673G>C	p.C558S
11	c.1702C>A	p.H568N	CCB	1	c.1704C>G/c.1703A>T	p.H568Q/p.H568L
12	c.1726G>T	p.G576C	CCB	1	c.1726G>C/c.1726G>A/c.1727G>A	p.G576R/p.G576S/p.G576D
13	c.1834C>A	p.H612N	CCB	2	c.1834C>T/c.1835A>C/c.1834A>G/c.1836C>G	p.H612Y/p.H612P/p.H612R/p.H612Q
14	c.1848C>A	p.D616E	CCB	5	c.1846G>A	p.D616N
15	c.1855A>C	p.S619R	CCB	3	c.1857C>G/c.1856G>A	p.S619N/p.S619R
16	c.1910T>C	p.L637P	CCB	1	c.1909C>A	p.L637M
17	c.1922T>G	p.L641R	CCB	1	c.1921C>G	p.L641V
18	c.1942G>C	p.G648R	CCB	4	c.1942G>A/c.1943G>A	p.G648S/p.G648D
19	c.1994G>A	p.G665E	CCB	2	c.1993G>A	p.G665R
20	c.2011A>G	p.M671V	CCB	1	c.2012T>G/c.2012T>A	p.M671R/p.M671K
21	c.2014C>G	p.R672G	CCB	1	c.2014C>T/c.2015G>T/c.2015G>A	p.R672W/p.R672L/p.R672Q
22	c.2021A>G	p.H674R	CCB	1	c.2020C>G/c.2020C>T/c.2022C>G	p.H674D/p.H674Y/p.H674Q
23	c.2461G>T	p.G821W	PB	1	c.2461G>A	p.G821R
24	c.2105G>C	p.R702P	CCB	1	c.2105G>A/c.2105G>T	p.R702H/p.R702H

Unpublished variants					Described in ClinVar or PD database, but without a referring publication	
1	c.49G>A	p.A17T	SP	1	ClinVar	
2	c.406T>A	p.Y136N	TF	2		
3	c.576G>T	p.E192D	NTB	1		
4	c.631G>A	p.V211M	NTB	3	ClinVar	
5	c.716T>C	p.L239P	NTB	1		
6	c.844G>T	p.D282Y	NTB	1		
7	c.1136C>A	p.S379Y	CCB	1	ClinVar	
8	c.1139C>T	p.S380F	CCB	1		
9	c.1174A>C	p.T392P	CCB	1		
10	c.1193T>C	p.L398P	CCB	1	ClinVar	
11	c.1292T>C	p.L431P	CCB	1		
12	c.1307G>A	p.R436Q	CCB	1		
13	c.1554C>G	p.D518E	CCB	1		
14	c.1744G>C	p.A582P	CCB	1		
15	c.2019C>A	p.N673K	CCB	1		
16	c.2558C>A	p.A853D	DB	1		
17	c.2570T>G	p.L857R	DB	1		

Abbreviations: CCB, catalytic (*β*/*α*)_8_ barrel domain; DB, distal *β*-sheet domain; NTB, N-terminal *β*-sheet domain; PB, proximal *β*-sheet domain; PD database, Pompe disease GAA variant database; SP, signal peptide; TF, trefoil domain.

**Table 2:** Comparison of early- versus late-onset PD cases.

	**Early-onset (** **n** **)**	**Late-onset (** **n** **)**
Total cases	458	265
Mean enzyme activity in PD cases (*μ*mol/L/h)	0.16 ± 0.23	0.35 ± 0.35
Homozygous cases	392 (85.6%)	64 (24.1%)
Compound heterozygous cases	66 (14.4%)	201 (76.9%)
Different *GAA* variants	196	142
Missense variants among them	81 (41.3%)	86 (60.6%)
Variants exclusively found in early-onset cases	144	0
Variants exclusively found in late-onset cases	0	90
Most frequent alterations found in early-onset cases		
c.896T>C/p.L299P	34	3
c.1314C>A/p.Y438⁣^∗^	29	0
c.236_246del11	19	0
c.2560C>T/p.R854⁣^∗^	18	7
c.877G>A/p.G293R	15	4
c.2237G>A/p.W746⁣^∗^	14	3
c.258dupC	13	2
c.1927G>A/p.G643R	12	5
c.1942G>A/p.G648S	12	4
Most frequent alterations found in late-onset cases		
c.-32-13T>G	9	167
c.525delT	5	17
c.2482_2646del (Ex18del)	2	12
c.307T>G/p.C103G	2	12
c.1655T>C/p.L552P	6	11
c.2015G>A/p.R672Q	3	9
c.784G>A/p.E262K	4	8

**Table 3:** Homozygous alterations in early- or late-onset cases.

	**Early-onset (** **n** ** =)**	**Late-onset (** **n** ** =)**
Different homozygous variants	150	41
Missense variants among them	60 (39%)	36 (87%)
Most frequent homozygous alterations found in early-onset cases		
c.896T>C/p.L299P	32	0
c.1314C>A/p.Y438⁣^∗^	27	0
c.2560C>T/p.R854⁣^∗^	17	2
c.236_246del11	17	0
c.877G>A/p.G293R	15	1
c.258dupC	12	1
c.1927G>A/p.G643R	11	0
c.1942G>A/p.G648S	10	1
c.2237G>A/p.W746⁣^∗^	10	0
Most frequent homozygous alterations found in late-onset cases		
c.2015G>A/p.R672Q	3	9
c.-32-13T>G	2	5
c.1979G>A/p.R660H	1	4
c.670C>T/p.R224W	2	4

## Data Availability

The data that support the findings of this study are available on request from the corresponding author. The data are not publicly available due to privacy or ethical restrictions.

## Supporting Information

Supporting Information 1 Figure S1: distribution of informative missense variants (a) and nonsense variants (b) along the GAA protein. Recurrent variants were excluded. The variants are expressed as a percentage. The most important information is highlighted in red. Abbreviations: SP, signal peptide; PP, propeptide; PB, proximal *β*-sheet domain; DB, distal *β*-sheet domain; N, number of variants;

Supporting Information 2 Table S1: four variants classified as variants of unknown significance (VUS) and therefore excluded. Table S2: unpublished truncation variants/small in-frame variants (*n* = 57). Table S3: GAA variants (*n* = 144) exclusively found in our IOPD cases. Table S4: GAA variants (*n* = 90) exclusively found in our LOPD cases.
