# Supplementary material for: Biochemical and Genetic Testing of GAA in Over 30.000 Symptomatic Patients Suspected to Be Affected With Pompe Disease
Source: Hum Mutat. 2024 Oct 22;2024:6248437. doi: 10.1155/2024/6248437 (PMC11918499; doi:10.1155/2024/6248437)
Supplement: Supporting Information 1 — Figure S1: distribution of informative missense variants (a) and nonsense variants (b) along the GAA protein. Recurrent variants were excluded. The variants are expressed as a percentage. The most important information is highlighted in red. Abbreviations: SP, signal peptide; PP, propeptide; PB, proximal β-sheet domain; DB, distal β-sheet domain; N, number of variants; [file 6248437.f1.docx]

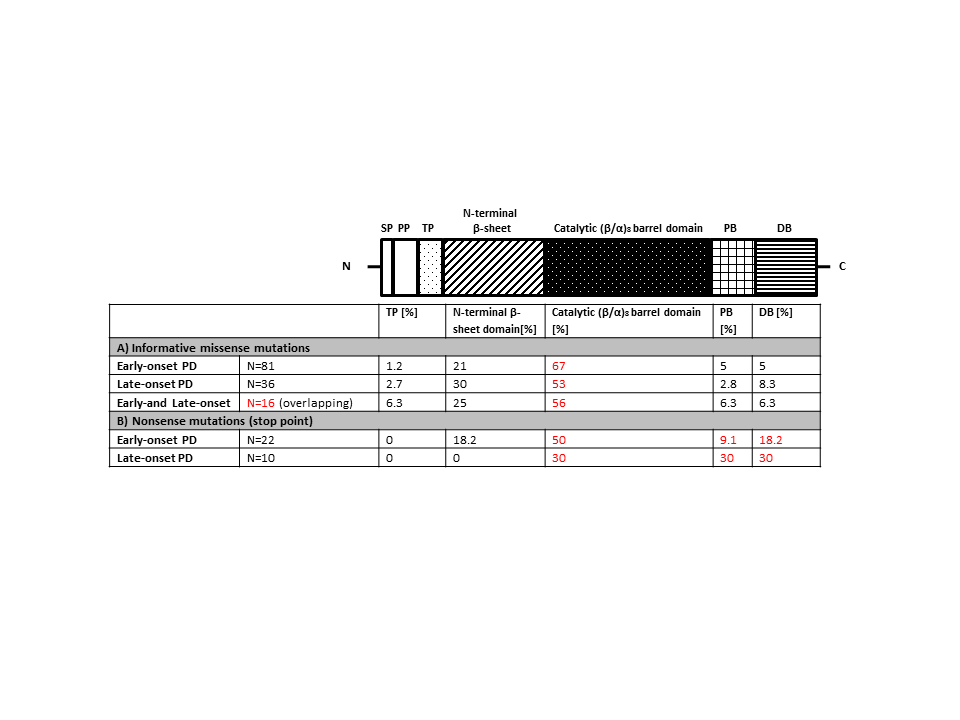


**Supplementary Figure S1: Distribution of informative missense variants (A) and nonsense variants (B) along the GAA protein.** Recurrent variants were excluded. The variants are expressed as a percentage**.** Most important information is highlighted in red. Abbreviations: SP-signal peptide; PP-propeptide; PB-proximal β-sheet domain; DB-distal β-sheet domain; N-number of variants;
